# Pressure Injury Risk Assessment in Nursing Practice: A Head-to-Head Comparison of the Braden Scale and Machine Learning Models

**DOI:** 10.3390/jcm15124683

**Published:** 2026-06-17

**Authors:** Fredy Barriga-Gallegos, Gonzalo Ríos-Vásquez, Paulo Figueroa-Torrez, Hanns de la Fuente-Mella, Catherine Almarza Garrido, Naldy Febré Vergara

**Affiliations:** 1 Institute for Health Care Research, Faculty of Nursing, Universidad Andrés Bello, Santiago 8370146, Chile; 2 Transportation and Logistics Center, Department of Engineering Sciences, Universidad Andrés Bello, Santiago 7500735, Chile; 3 School of Industrial Engineering, Pontificia Universidad Católica de Valparaíso, Valparaíso 2340025, Chile; gonzalo.rios@pucv.cl; 4 Departamento de Ciencias Industriales, Medio Ambiente y Energía, Universidad Católica Boliviana San Pablo, Colón 734, Tarija, Bolivia; pfigueroa@ucb.edu.bo; 5 Instituto de Estadística, Pontificia Universidad Católica de Valparaíso, Valparaíso 2340031, Chile; hanns.delafuente@pucv.cl; 6 Subdirection of Care Management, Félix Bulnes Clinical Hospital, Santiago 9110056, Chile; c.almarzagarrido@uandresbello.edu

**Keywords:** pressure ulcers, machine learning, Braden scale, health risk evaluation, risk stratification, clinical decision support

## Abstract

**Background**: Pressure injury (PI) prevention relies heavily on the application of the Braden Scale, yet its predictive performance is limited mainly due to subjective measurements and fixed cut-off points, yielding potential errors in terms of false-positive alarm rates and workforce misuse. Machine learning (ML) has emerged as a promising approach to improve discrimination, but fair comparisons with traditional tools remain scarce in clinical settings. **Methods**: Using data from 446 hospitalized patients in a tertiary hospital in Chile, we compared five classic ML models for classification against the Braden Scale. ML models were trained exclusively on routinely collected clinical and nursing variables, excluding Braden inputs. A matched operating-point framework was applied, aligning ML decision thresholds with Braden cutoffs based on equivalent recall or specificity. **Results**: The XGB model showed the highest overall discrimination performance (AUC = 0.835). When matched to Braden recall, XGB achieved about 17% gains in specificity, substantially reducing false positives. When matched on specificity, recall improvements ranged from 13% to 25%. These gains were consistent across clinically relevant thresholds considered during the comparison. **Conclusions**: ML models, particularly XGB, outperform the Braden Scale under equivalent clinical operating conditions. Rather than replacing Braden, ML has emerged as a promising approach that preserves patient safety while improving precision and resource allocation.

## 1. Introduction

According to the National Pressure Injury Advisory Panel (NPIAP), a pressure injury (PI) is localized damage to the skin and/or underlying soft tissue, typically occurring over a bony prominence or in relation to a medical or other device, resulting from intense and/or prolonged pressure or pressure in combination with shear. The injury can present as intact skin or an open ulcer and may be painful [1].

PIs represent a major patient safety concern and a persistent challenge for healthcare systems worldwide due to their high prevalence, morbidity, and impact on healthcare resources. It remains highly prevalent among hospitalized adult patients globally, with substantial variability across regions and care settings, underscoring their widespread clinical burden [2].

This burden is particularly pronounced in intensive care units (ICUs), where patients are exposed to multiple risk factors, including immobility, hemodynamic instability, the use of medical devices, and the severity of illness. Patients in critical care are at higher risk of developing PIs than those in the general ward [3].

Beyond their incidence, PIs are associated with adverse clinical and economic outcomes, including prolonged hospital stays, increased nursing workload, higher healthcare costs, and reduced health-related quality of life. Hospital-acquired pressure injuries have been linked to substantial increases in direct medical costs, with national estimates in the United States reaching several billion dollars annually [4]. At the patient level, PIs contribute to pain, functional limitations, psychological distress, and diminished well-being [5]. Together, these consequences underscore the importance of effective risk assessment, prevention, and early intervention strategies, particularly in high-risk settings such as intensive care units [6].

The development of PIs is multifactorial. Patient-related factors such as advanced age, impaired mobility, comorbidities, and reduced physiological reserve have been consistently associated with an increased risk across healthcare settings [3]. In acute and critical care contexts, this vulnerability is further exacerbated by care-related factors, including prolonged immobility, hemodynamic instability, and the use of vasoactive agents, which compromise tissue perfusion and tolerance [7,8]. In addition, moisture-related factors, particularly urinary and fecal incontinence, as well as pressure and shear generated by medical devices, have been robustly linked to PI development, especially in ICU populations [9,10].

To support clinical risk assessment, several tools have been proposed, among which the Braden Scale is one of the most widely used instruments for hospitalized patients. Despite its widespread adoption, numerous studies have reported limitations in its predictive performance, particularly its reliance on fixed cut-off points to classify patients as at risk. Evidence from intensive care settings indicates that commonly used thresholds, such as 18 or 16, exhibit limited predictive validity and poor precision, restricting the scale’s ability to discriminate between patients who will and will not develop pressure injuries [11]. This limited discrimination often results in high false-positive rates and the potential overuse of preventive resources [12]. Moreover, optimal cut-off points vary substantially across clinical contexts, challenging the applicability of a single universal threshold. Although modified versions of the Braden Scale have been proposed, these adaptations have not fully resolved its fundamental limitations in predictive accuracy [13]. Consequently, while the Braden Scale remains useful as a conservative screening tool for general risk stratification, its capacity to accurately predict PI development at the individual patient level is inherently limited.

These limitations have motivated increasing interest in data-driven approaches. Unlike rule-based scoring systems, machine learning (ML) models can integrate a large number of clinical variables and capture nonlinear relationships among patient characteristics, care-related factors, and clinical context. In recent years, a growing body of literature has explored ML-based methods for PI prediction, generally reporting improved discrimination and predictive accuracy compared with traditional risk assessment tools [14,15,16,17,18,19,20]. Most proposed models rely on demographic, clinical, and care-related variables extracted from electronic health records and employ a wide range of algorithms, from classical methods such as logistic regression and decision trees to ensemble and deep learning approaches [21,22,23,24,25].

Several authors have incorporated the Braden Scale, either as a total score or through selected subscales, as an input feature rather than as a standalone risk stratification tool, reflecting its clinical relevance but also its limited performance when used in isolation [26,27]. Other works have explored alternative data modalities, including nursing notes, sensor-derived physiological signals, and medical device-related information, highlighting the potential of ML approaches to capture complex and context-specific risk patterns [28,29]. However, many existing models remain constrained by retrospective designs, limited external validation, or reliance on features that are not routinely available in nursing workflows.

Despite the widespread use of the Braden Scale and the growth of ML-based approaches for PI prediction, there is limited systematic evidence on the clinical viability and operational performance of the Braden Scale itself, compared with statistical and ML models under equivalent decision conditions. Most existing studies either use Braden-derived variables as input features or report performance comparisons at arbitrarily selected thresholds without explicitly accounting for the trade-offs between recall and specificity that guide clinical screening decisions. Consequently, the extent to which ML models truly outperform standard risk stratification, beyond threshold effects, remains insufficiently characterized. To address this gap, the present study compares the Braden Scale with multiple ML models using a matched operating-point framework. By explicitly preserving the screening properties of the Braden Scale while assessing gains in discrimination, this work provides novel evidence on how data-driven models can complement, rather than replace, established nursing risk assessment tools.

### Research Questions and Hypotheses

Based on the limitations identified in traditional pressure injury risk assessment tools and the increasing adoption of ML approaches in clinical prediction, this study aims to evaluate the differences in the predictive performance of ML tools over the Braden Scale under equivalent clinical operating conditions. Accordingly, the following research questions and hypotheses are proposed for this study.

- **RQ1**. Do ML models outperform the Braden Scale in predicting PI under equivalent clinical operating conditions?
- **RQ2**. Can ML models reduce false-positive classifications while maintaining clinically acceptable sensitivity compared with the Braden Scale?
- **RQ3**. Does the Braden Scale retain clinically meaningful predictive performance for PI risk assessment when evaluated under multiple risk cutoffs?

Furthermore, the following hypotheses are proposed:

**Hypothesis** **1.** *ML models achieve significantly higher discrimination performance than the Braden Scale when evaluated under matched operating conditions*.

**Hypothesis** **2.** *When calibrated to equivalent levels, ML models yield superior complementary performance metrics compared with the Braden Scale*.

**Hypothesis** **3.** *The Braden Scale has clinically relevant predictive performance across established risk thresholds, supporting its continued use as a conservative screening tool in nursing practice*.

## 2. Literature Review

The reliance on traditional risk scores in health assessments raises concerns about a wide range of issues. Authors claim that estimated risks can be unreliable and subjective, resulting in poor accuracy that affects the health-related quality of life of patients, along with the misuse of resources in health settings [30].

Failure to recognize and respond to patients in a timely manner has been identified as an international patient safety concern. Several studies have shown deficiencies in assessing adverse events occurring as a result of failing to recognize acute risk events in a timely manner. For instance, authors have shown limitations in the National Early Warning Score for deteriorating adult patient assessment, mainly due to poor risk judgment, which yields delayed responses that lead to unplanned intensive care admissions [31,32]. Other studies have analyzed the limitations of Cardiovascular Risk Scores that restrict their applicability and impact their performance [33,34,35]. In terms of PI risk assessment, the Braden Scale has been analyzed with respect to its predictive validity. A meta-analysis concluded that the Braden Scale’s predictive validity is moderate and that it can over-label the risk of patients [36]. Other studies have concluded that it presents heterogeneity across studies and that cutoffs differ by context in terms of demographic characteristics. Thus, calibration is a real issue in health settings [37,38,39].

In this regard, several studies have compared the discrimination ability of traditional scales against advanced modeling tools (e.g., statistical learning, ML, deep learning). For instance, researchers have found that ML models outperform traditional malnutrition scores, yielding better identification performance [40,41]. Other studies have done the same in terms of fall risk scores (e.g., Hendrich II) [42,43], deterioration scores (e.g., MEWS and NEWS 2 scores) [44,45], and PI scores for patients in intensive care units [46]. All of them show that advanced modeling yields better capabilities for risk assessments, serving as complementary tools for early risk assessments and improving the health-related quality of patients during hospital stays.

Beyond the advantages of computational algorithms over traditional scales, ML models are typically used to estimate the probability that a patient will develop a PI based on a set of features of interest. Furthermore, such probability is commonly seen as a risk measure that allows nursing staff to pay attention to certain individuals based on the results yielded by the models employed [25]. Overall, the approaches to estimate the risk and probabilities of PI development range from interpretable models and ensemble-based methods to more advanced methods based on neural networks [47]. In practical terms, ML-based tools support nursing practice and serve as complementary approaches to traditional risk scales such as the Braden Scale. However, their clinical value depends on data preprocessing, validation, calibration, interpretability, and integration capabilities into existing workflows [48].

Given the central role of the Braden Scale in both routine nursing practice and the development of data-driven prediction models, a clear understanding of its structure, scoring system, and clinical use is essential for interpreting subsequent methodological choices and comparative analyzes.

### 2.1. Braden Scale

As stated above, the Braden scale is a tool used for assessing the risk of pressure injuries. It is widely used to estimate a patient’s likelihood of developing pressure injuries. It is designed to be simple, quick, and usable at the bedside, mainly by nursing staff [37]. It is based on the skin’s tolerance to recover when pressure, shear, moisture, and impaired tissue tolerance exceed certain thresholds. The Braden scale attempts to operationalize these mechanisms into six domains that can be understood in clinical settings, each of which is scored based on clinical judgment. The domains are shown in Table 1 with details of what they measure. Each domain is scored from 1 (worst) to 4 (best), except for friction and shear, which are scored from 1 to 3, comprising a total score range of 6 to 23 [49].

In clinical settings, the typical cutoffs of the Braden Scale that are used internationally comprise the following: very high risk (≤9), high risk (10–12), moderate risk (13–14), mild risk (15–18), and low or no risk (>18) [11].

Even with emerging tools for risk assessment (e.g., statistical learning, computational algorithms), Braden remains popular mainly due to its low cost, fast implementation (less than 5 min), no technology required, clinical interpretability, and because it is embedded in nursing workflows. Despite its popularity, it shows several limitations, especially among borderline-risk patients [50].

While the structure and scoring of the Braden Scale are internationally standardized, its clinical implementation and operational use may vary across healthcare systems and national guidelines.

### 2.2. Braden Scale in Chilean Context

In Chile, the Braden scale is explicitly recognized and endorsed within the national framework for PI prevention by the National Ministry of Health (MINSAL). Braden is included in technical norms and serves as guidance on PI prevention for healthcare facilities. Additionally, it is regarded as a standard risk-screening tool for various patient types. It is important to note that Braden is part of quality-of-care and patient safety strategies. The Chilean norm endorses its systematic use combined with preventive nursing interventions, which means that there is no mandated single score cutoff. This latest denotes its role as a complementary risk assessment tool for patient care [51].

In practice, Chilean health centers operationalize Braden through local protocols, typically stating that Braden must be applied during patients’ admissions by nursing staff. When there is clinical deterioration, transfer to other wards, or during regular intervals, reassessments of Braden risk are conducted. Usually, scores are recorded in the nursing record linked to preventive bundles [52].

In summary, the Braden scale is a nurse-administered clinical risk assessment tool that evaluates six domains related to PI development. In Chile, it is formally endorsed by national health authorities and deeply embedded in hospital protocols as a standard screening instrument. This institutional role makes the Braden Scale a natural clinical benchmark for evaluating alternative risk prediction approaches, particularly in the context of data-driven models applied to routinely collected nursing information.

## 3. Methods

This section describes the study design, data sources, preprocessing procedures, ML model development, and evaluation framework used to compare data-driven prediction models with the Braden Scale.

Data preprocessing and ML model development were performed using Python 3.13.9. All preprocessing procedures were carried out using Scikit-learn Pipelines through an out-of-fold approach, ensuring that each transformation was fitted solely with training fold features and applied over testing folds, thereby avoiding potential data and target leakage.

### 3.1. Study Design and Data Source

This study employed an observational cross-sectional design, using data obtained from a tertiary-level hospital located in an urban area of Santiago, Chile. The sample size was estimated assuming a 95% confidence level, a 5% margin of error, and an anticipated 20% loss, resulting in an initial target of approximately 500 patients. Because no prior local prevalence estimates were available for the target population at the time of study design, a conservative proportion assumption was adopted to maximize sample size requirements. Data were obtained through convenience sampling by healthcare professionals across different hospital wards. Eligible participants included hospitalized patients with available nursing and clinical assessment records collected during routine inpatient care. Data collection was conducted between January and December 2022 across multiple hospital wards at Félix Bulnes Clinical Hospital in Santiago, Chile. The collection process was conducted by trained healthcare professionals involved in routine patient care and institutional prevalence-monitoring activities. Standardized data collection forms and patient medical records were used to ensure consistency in the registration of clinical and nursing variables across hospital units. Records presenting duplicated entries, inconsistent identifiers, clinically implausible observations attributable to data-entry errors, or missing outcome information regarding PI status were excluded from the analysis. After data cleaning and preprocessing, the final analytical sample included 446 patients.

### 3.2. Data Collection

The data collection forms were previously reviewed and validated by a panel of clinical experts to ensure consistency in the interpretation and categorization of nursing and clinical variables. This process included standardizing free-text annotations and shorthand clinical notations into structured, clinically meaningful categories. Because hospital information systems may contain multiple records per patient, an aggregation and conflict-resolution procedure was implemented during data preprocessing. Clinically inconsistent or implausible values were reviewed and corrected when possible, while duplicate observations were removed prior to analysis. For categorical variables, conflicting entries were resolved using clinically conservative criteria, prioritizing the most severe reported condition.

The instruments captured patient-related characteristics and factors associated with PI development. The final dataset comprised four domains: (i) demographic characteristics, (ii) clinical parameters at admission, (iii) laboratory and diagnostic tests requested by the treating physician, with results available within 24 h of admission, and (iv) PI-related variables, including risk assessment, preventive interventions, and severity of tissue damage. Dependency risk was assessed using the Clasificación de Riesgo–Dependencia (CUDYR), a validated nursing instrument adopted by the Chilean Ministry of Health, which classifies hospitalized patients according to their level of care dependency (categories A–D, from highest to lowest).

### 3.3. Outcome Definition

PI status was defined according to the National PI Advisory Panel (NPIAP) criteria and treated as a binary outcome. Patients were classified as PI-positive if at least one lesion was identified at any anatomical site, regardless of stage (Stage 1–4 or suspected deep tissue injury), and as PI-negative otherwise [53]. Cases with incomplete or indeterminate PI outcome classification were not considered in the final analytical dataset.

### 3.4. Predictor Variables and Temporal Validity

Predictor variables were selected from routinely collected clinical and nursing information available during standard inpatient care. No variables reflecting information obtained after the PI occurred were included, ensuring that model predictions were based on data available prior to the outcome and preventing temporal information leakage, thereby allowing a fair comparison between ML models and the Braden scale in equivalent clinical conditions. The Braden Scale was excluded from the feature set used to train the ML models and was evaluated separately as a standalone comparator. Model performance, therefore, reflects predictions based solely on clinical variables without incorporating prior risk stratification.

### 3.5. Experimental Setting

**Figure 1:**
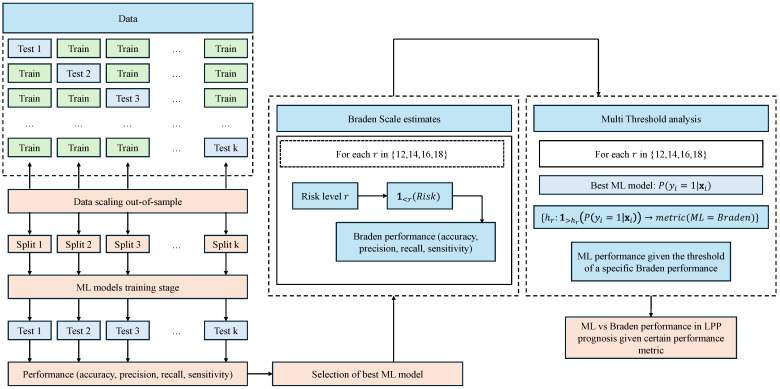
Steps conducted in the experimental setup for fitting ML models and comparing performance with the Braden scale in matched-operating points.

Figure 1 shows the experimental setup used in the analysis. Each step is detailed in the following paragraphs.

#### 3.5.1. Data Preprocessing

Relevant nursing documentation was extracted from the Hospital Information System (HIS) for each patient record. From this source data, key clinical concepts associated with PI development were identified and organized into a structured dataset. This process was conducted in collaboration with clinical experts to ensure the semantic consistency and clinical validity of the extracted variables.

A predefined set of demographic, clinical, nursing care, device-related, and risk assessment variables was selected. Records with missing values in the outcome variable, duplicated entries, or implausible observations attributable to data entry inconsistencies were excluded prior to model development. Implausible values due to unit inconsistencies (e.g., body weight recorded in grams) were corrected, and zero values in weight, height, and age were treated as missing because these measurements are clinically impossible and were interpreted as data-entry or registration errors rather than true observations.

Missing categorical values were imputed using the mode, while continuous variables (weight, height, age, and waiting time) were imputed using group-wise mean values stratified by clinical service and gender. Group-wise imputation preserves local clinical variability across hospital services and patient profiles. This strategy was adopted to preserve the sample size and avoid excluding clinically relevant patient records, given the moderate dataset size. The remaining missing height values were imputed using the overall mean, and extreme age values were excluded as data entry errors.

#### 3.5.2. Predictor Inclusion Strategy

All available predictors were retained for model development. No feature selection or dimensionality reduction techniques were applied, as the objective of this study was to compare ML models with the Braden scale under equivalent clinical information. This approach avoids introducing selection bias and preserves the full set of clinically relevant variables.

#### 3.5.3. Cross-Validation Process

To ensure model validation in a reliable and reproducible way, a cross-validation process is conducted during the fitting procedure of the ML models. This step addresses the generalization of results, ensuring the statistical properties of the performance through the generated folds. The setting considered for this step is a *k*-fold cross-validation technique, which trains the model using k−1 folds of the data and tests the performance in the remaining fold. This process is repeated *k* times, yielding *k* performance metrics for each split of the data. The same testing folds generated during the five-fold cross-validation procedure were used to evaluate both the ML models and the Braden Scale, ensuring that all comparisons were conducted on identical patient subsets. This generates a distribution of the performance, allowing for a robust analysis instead of point estimates of the error metrics [54].

### 3.6. Machine Learning Models

Five supervised ML models for binary classification were evaluated, considering various paradigms of statistical learning: for tree-based models, a Decision Tree (DT), a Random Forest (RF), and Extreme Gradient Boosting (XGB) were fitted. A Logistic Regression (LR) was used for logit models. A margin-based model considering Support Vector Machines for Classification (SVC) was employed. This setup allows for the assessment of both linear and nonlinear relationships that might be present in the data. All models were implemented using the Scikit-learn library version 1.7.2 [55], while XGB was implemented using the XGBoost framework.

### 3.7. Model Training and Hyperparameter Optimization

Prior to model training, categorical variables were one-hot encoded, and numerical variables (weight, height, age, and waiting time) were standardized using z-score normalization (mean centering and unit variance scaling) via the StandardScaler class. To prevent data leakage, scaling parameters were estimated exclusively on the training data within each fold and subsequently applied to the corresponding test data.

Model evaluation was conducted using five-fold cross-validation. For each algorithm, an exhaustive grid search over predefined hyperparameter ranges was performed, with model performance assessed independently within each fold. Table 2 summarizes the specific hyperparameters explored for each model and their corresponding value ranges. Hyperparameter tuning was conducted to appropriately calibrate model complexity, as predictive performance depends not only on data quality but also on adequate parameter configuration. This process contributes to improving model robustness, generalizability, and clinical utility.

### 3.8. Class Imbalance Handling

Class imbalance was addressed using class-weighted loss functions when supported by the algorithm (DT, LR, RF, and SVC). This approach penalizes the misclassification of the minority class without introducing synthetic samples, which is particularly appropriate for moderate sample sizes. No resampling techniques were applied.

### 3.9. Evaluation Metrics

Model performances were evaluated using metrics derived from the confusion matrix for a binary classification setting. For threshold-dependent variables, accuracy, precision, recall (sensitivity), and specificity were computed. Furthermore, the receiver operating characteristic curve was computed for the best model to check performance with various thresholds for classification. Given the *k*-folds cross-validation setting, the minimum, maximum, and mean values of each model were recorded for the metrics considered. This allows for accounting for the variability and robustness of each configuration, yielding a transparent comparison among models.

Given that various metrics were recorded during the fitting procedure for each model, a composite metric based on the geometric mean score (GMS) of the various performance metrics is calculated for each combination of hyperparameters. Its calculation is shown in the Supplementary Material.

This setting allows for selecting the hyperparameters in a robust way compared with other metrics that can be calculated, such as the arithmetic mean. Note that the multiplicative effect of the GMS means that if a model performs poorly on one metric, it causes the GMS to yield poor composite performance for a specific model configuration. Later, the selected model configuration is the one that yields the best performance according to the computed GMS.

### 3.10. Matched Operating-Point Comparison with the Braden Scale

ML classifiers output the probability that an instance belongs to a particular class. In the context of this study, the model provides the probability of PI occurrence for an individual, given a set of features used to calculate that risk. More details about this calculation and threshold rules can be found in the Supplementary Material.

The Braden scale yields an ordinal risk score, and clinical practice defines risk categories by applying established cutoffs, each of which induces a specific trade-off between recall (sensitivity) and specificity. Validation studies of the Braden scale support its use as a clinically meaningful baseline for PI risk stratification [50]. Consequently, comparing methods at an arbitrary threshold (e.g., t=0.5 for ML) may lead to biased conclusions driven by threshold choice rather than true discriminative ability. Consistent with this design, the Braden Scale was not used as an input feature in any ML model.

To ensure a fair and clinically meaningful comparison, we adopted a matched operating-point comparison based on clinically constrained performance metrics [56]. The central idea is to constrain a primary metric (or a pair of clinically relevant metrics) to be comparable across methods and then compare all other metrics under that constraint. This approach aligns with the view that the evaluation of clinical prediction models should reflect clinically interpretable operating conditions rather than relying on single metrics or arbitrarily chosen thresholds [57]. Accordingly, models were compared at equivalent clinical operating points defined by recall and specificity rather than at arbitrarily chosen thresholds.

The procedure was implemented as follows:

1. **Outcome definition:** The clinical outcome was defined as the presence or absence of PI, resulting in a binary target variable.
2. **Baseline clinical tool:** The Braden scale was used as the baseline clinical tool, producing an ordinal risk score for each individual. Clinically accepted cutoffs were used to define operating points characterized by known recall–specificity trade-offs.
3. **Selection of clinically relevant operating points:** Multiple Braden thresholds were selected a priori. Each threshold corresponds to a distinct operating point, defined by a specific pair of recall and specificity values. As shown in Figure 1, four operating points are set, specifically {12,14,16,18}. This selection is mainly based on the risk-profiles each category yields. Patients with a Braden score lower than 12 are denoted as having high risk profiles. A score between 13 and 14 is considered moderate risk. A Braden score between 15 and 18 indicates mild risk, so 16 is selected as the mid-point. Finally, a Braden score of 18 or higher indicates no risk.
4. **Construction of comparable ML operating points (threshold matching):** For each Braden operating point, an ML probability threshold $h^{*}$ was selected such that the resulting recall and specificity were approximately equal to those obtained with the corresponding Braden cutoff. This step constitutes a matching procedure between Braden and ML decision thresholds and not a threshold optimization process.
5. **Comparison under equal constraints:** Given comparable recall and specificity, secondary performance metrics (accuracy, precision, AUC, and any remaining metrics not used as constraints) were compared across methods. This design ensures patient safety (recall) while controlling the operational burden associated with false-positive alerts (specificity). Under this framework, observed differences reflect discriminative performance rather than arbitrary threshold choices.

## 4. Results

The prevalence of PI was about 19% considering all medical wards. The average age of patients was 47 years. About 42% of patients belonged to the adult surgical medical unit, followed by adult surgery at about 12%, and the emergency department at 8%. More than half of the patients were males (55%). About 5% of the patients entered the medical wards with a PI pre-hospital, and about 6.5% were admitted with skin lesions. Other important aspects to note are that approximately 38% of the patients had some form of incontinence, while 8% had mobility issues. Detailed descriptive statistics of predictor variables and the distribution of patients across hospital wards are provided in Supplementary Tables S1–S3.

**Figure 2:**
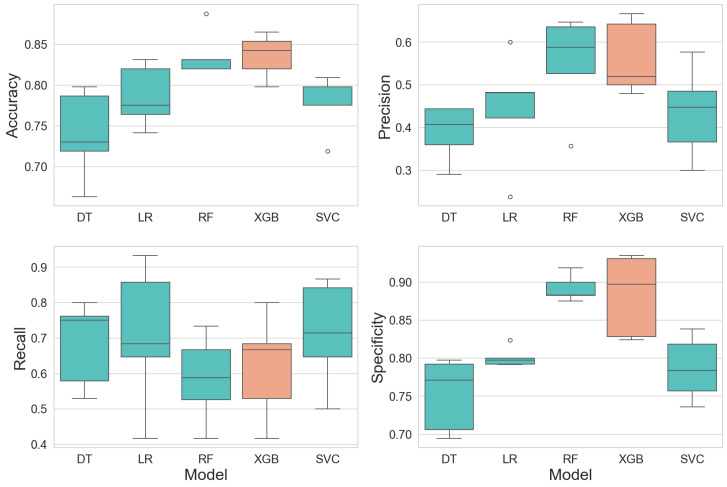
Performance metrics of ML models for a binary classification task during the cross-validation process.

Results of the performance metrics for the models employed are shown in Figure 2. In terms of accuracy, the XGB model showed the highest median accuracy with a tight interquartile range (IQR). The RF model was stable, with a narrow spread in its performance distribution. The SVC model was in the middle among the performances, with some low outliers, while the DT model was the weakest model with unstable results.

Regarding precision, the XGB and RF models were the strongest, with the latter showing higher median precision and greater variability. The SVC showed the widest performance distribution, while the DT had the worst performance among the models. In this sense, the XGB and RF models were best at avoiding false positives, aligning with the goals of workload reduction in prediction tools.

In terms of recall, the LR and SVC models had higher median performance. The XGB showed moderate performance with a similar median compared with the SVC and LR models. The RF showed the worst performance, with the lowest median.

Finally, regarding specificity, the XGB model dominated the performances with the highest median and the widest variability. The RF model followed with stable results. The DT model showed the worst performance, with the lowest median and a wide IQR of performance.

Table 3 shows a summary of the performance metrics, considering the GMS score calculated for the models across various classification metrics. The XGB and RF models showed the highest GMS, with the XGB exhibiting a slightly higher score, mainly due to better recall compared with the RF. The DT, LR, and SVC models showed similar results regarding GMS, with variability in terms of the best performing metrics.

Table 3 summarizes the average cross-validation performance of the evaluated ML models across standard classification metrics, as well as the composite GMS used for model selection.

**Figure 3:**
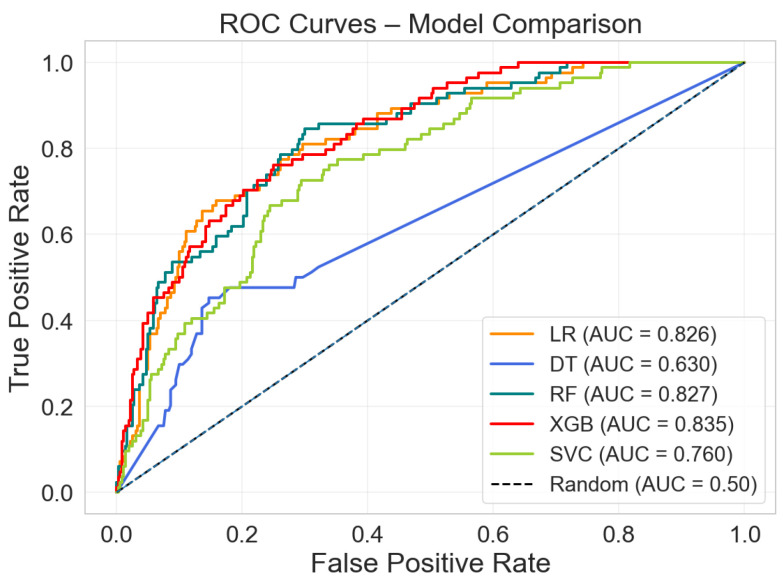
Average receiver operating characteristic curves for ML models during testing procedure. The diagonal line denotes a model that makes classification at random.

Figure 3 displays the ROC-AUC plots obtained from the cross-validation testing folds for the evaluated ML models. The XGB model showed the highest AUC (0.835), denoting the best overall discrimination. The RF and the LR showed similar performance, with an AUC of approximately 0.83. The SVC yielded moderate discrimination, while the DT showed poor discrimination ability, barely above random.

Given the results shown in Figure 2, the XGB model demonstrates better performance in terms of reducing false alarms while maintaining an acceptable recall. In this setup, the strength of XGB becomes clinically meaningful, providing a high-specificity triage option compared with the other models employed.

### Comparison Between Braden and ML Models

**Figure 4:**
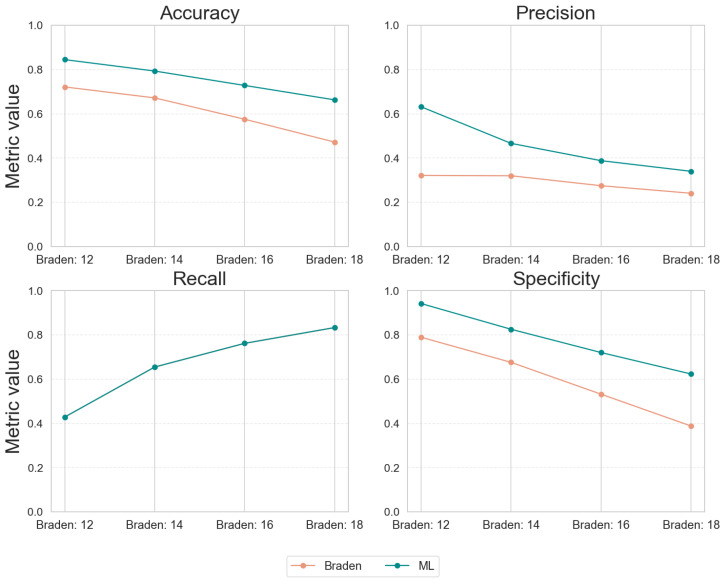
Prediction performance metrics of Braden Scale and XGB model matching recall as baseline for multi-threshold comparison.

The comparison of Braden against ML models in PI prognosis performance was developed by considering recall and specificity as the baselines to compute other metrics. Figure 4 shows the performance of the Braden and ML models matching recall for both approaches. The XGB model had adjusted its decision threshold to achieve the same recall as the Braden cutoffs. The results show that once XGB was calibrated to achieve a recall equivalent to the Braden scale at each cutoff, it consistently demonstrated higher specificity, precision, and overall accuracy. At identical recall levels, specificity gains ranged from 15 to 20% points, indicating a substantial reduction in false-positive classifications without increasing missed PIs.

These findings suggest that ML-based risk stratification can preserve the screening performance of the Braden scale while substantially reducing unnecessary preventive interventions.

**Figure 5:**
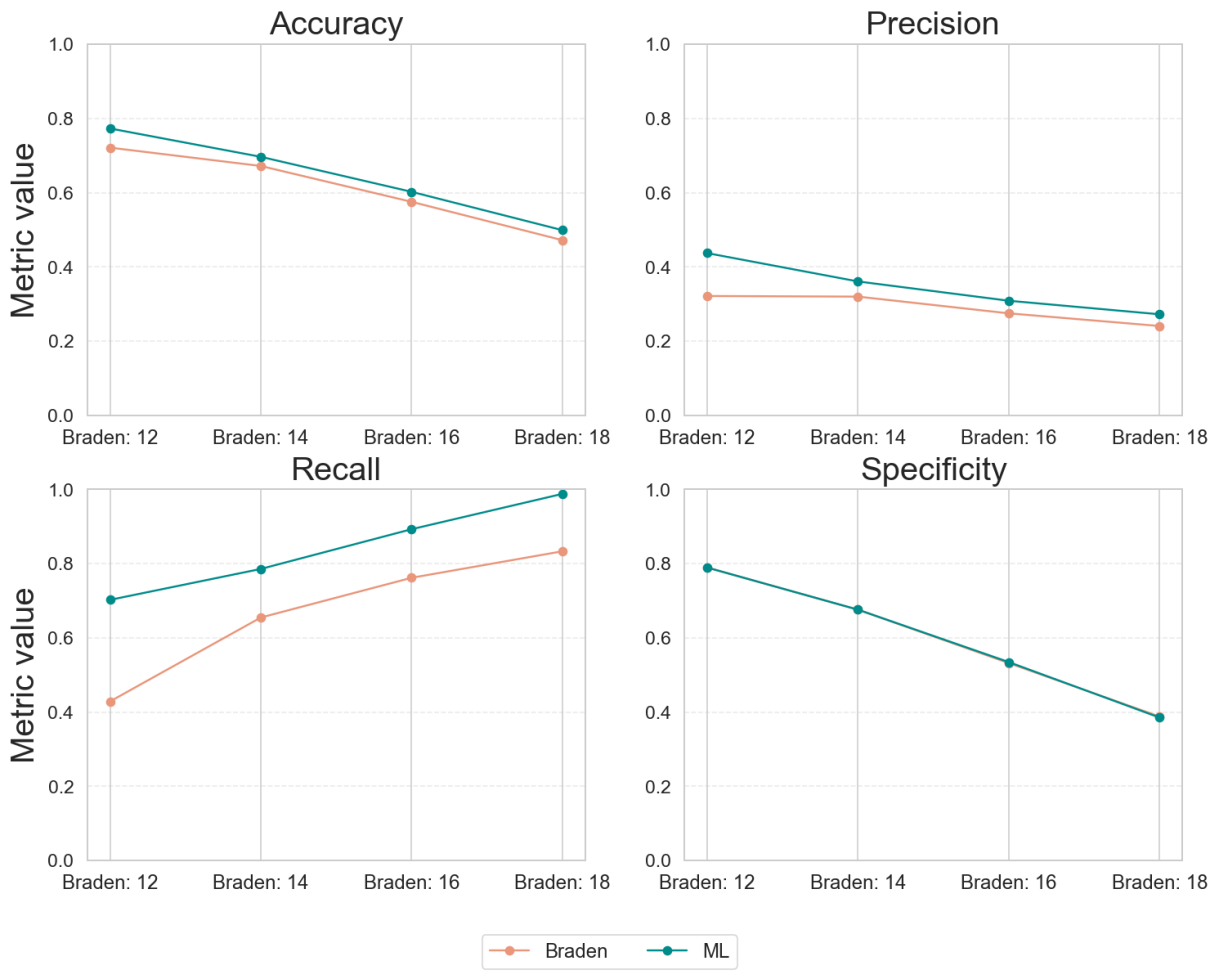
Prediction performance metrics of Braden Scale and XGB model matching specificity as baseline for multi-threshold comparison.

Figure 5 shows the results once Braden and ML are matched, considering specificity as the baseline metric. This addresses a different clinical question related to the equality of safety in ruling out low-risk patients with different detection methods. Once XGB was calibrated, considering similar specificity to the Braden scale, it consistently demonstrated higher sensitivity across all cutoffs, with recall improvements ranging from 13 to 25% points. Precision and overall accuracy were also higher for the ML model at matched specificity.

Taken together, sensitivity-matched and specificity-matched analyzes indicate that the ML model consistently outperforms the Braden scale across clinically relevant operating points.

Overall, these findings provide evidence supporting the research hypotheses. The results indicate that ML models, particularly XGB, outperform the Braden Scale under equivalent clinical operating conditions while preserving clinically relevant screening performance. At the same time, the Braden Scale demonstrated meaningful standalone predictive performance across multiple cutoffs, supporting its continued clinical relevance as a risk assessment and screening tool in nursing practice.

## 5. Discussion

The present study showed that ML models, particularly XGB, achieved superior discrimination performance compared with the Braden Scale under matched operating conditions. Furthermore, the XGB reached a better reduction in false-positive classifications while preserving clinically acceptable performance, specifically in terms of recall. These findings are consistent with previous literature reporting improved predictive performance of ML approaches for PI risk assessment compared with traditional clinical scores [15,16,18]. Furthermore, similar improvements in discrimination ability have also been reported in ML-based prediction systems beyond PI risk assessment [40,41,42]. However, unlike several previous studies that relied on fixed or specific classification thresholds, the present work evaluated ML and the Braden Scale under equivalent clinical operating conditions. This distinction is important because threshold selection can substantially influence apparent model superiority [58]. Therefore, the observed performance gains in this study reflect genuine improvements in discrimination ability rather than threshold-related artifacts with biased results. Importantly, the acceptable performance of the Braden scale under matched operating conditions reinforces its continued clinical relevance, especially in settings where transparency, interpretability, and ease of implementation are prioritized. This latest result supports the hypothesis that the Braden Scale retains clinically meaningful predictive utility despite being outperformed by ML models in overall discrimination performance.

Regarding clinical fairness, by working with metrics as a baseline, the comparison avoids overstating ML performance due to more aggressive thresholding, which aligns the evaluation with real-world clinical decision constraints [59,60]. Furthermore, the Braden scale’s stable performance suggests that expert-designed scores capture a strong signal, even with constrained flexibility. This explains why such tools remain adopted in clinical practice [37,61].

In this sense, ML adds value through systematic gains in classification performance, indicating better discrimination among borderline-risk patients. This is particularly useful where clinical resources are limited, and proper prioritization benefits both patients and the workforce [62].

It is worth mentioning that the results do not suggest a replacement or removal of the Braden Scale. Rather, ML models may be positioned as decision-support extensions, improving efficiency without replacing established clinical workflows. In addition, as the Braden Scale captures clinically relevant information, it may also serve as an important complementary input for statistical learning models [18,63].

From an implementation standpoint, the proposed ML framework could be integrated into information systems as an automated background-support tool. As the models rely exclusively on routinely collected variables available during admission, risk probabilities could be generated without requiring additional procedures by nursing staff. In practice, such a system could automatically classify patients into risk categories aligned with existing Braden-based protocols and alert healthcare professionals when elevated risk is detected [60]. Such integration would preserve existing workflows while enhancing precision in risk stratification and resource allocation.

Successful adoption of ML-based stratification, nevertheless, depends not only on predictive performance but also on usability, transparency, calibration, and alignment with institutional care protocols [59]. By positioning ML as an augmentation layer rather than a replacement mechanism, the transition toward data-driven support tools becomes more clinically sustainable.

In this sense, the ML model may complement Braden by refining risk within intermediate categories or by serving as a second-layer triage mechanism when clinical uncertainty exists [61]. Such hybrid approaches preserve the interpretability of Braden while leveraging the enhanced discrimination capacity of data-driven models. The feasibility of ML implementation depends not only on statistical performance but also on usability, transparency, and alignment with established care protocols. By positioning ML as an augmentation layer rather than a replacement mechanism, the transition toward data-driven support tools becomes clinically sustainable.

The findings also contextualize previous literature on the substantial performance gains of ML models over traditional risk scores. Several papers reported greater discrimination of ML-based approaches than conventional clinical scores, often relying on fixed or arbitrarily selected decision thresholds [14,16]. Threshold choices can amplify apparent differences in performance and lead to misleading conclusions if clinically relevant trade-offs are not explicitly considered [59,60]. By contrast, the matched operating-point framework adopted in this paper provides evidence that even when recall and specificity are explicitly constrained to reflect comparable clinical decision conditions, ML models retain a consistent advantage in discrimination. This indicates that the observed performance gains cannot be attributed solely to threshold choice but rather to substantive differences in how patient risk heterogeneity and nonlinear relationships are modeled.

The use of recall and specificity as matching constraints reflects the underlying decision to prevent PI. High recall prioritizes patient safety by minimizing missed cases, while specificity directly relates to workload and resource utilization. This trade-off has been explicitly discussed in the context of PI risk assessment and nursing decision-making, where conservative screening strategies are favored to ensure early identification of at-risk patients [4,50]. Methodology has emphasized that model evaluation should be anchored to clinically meaningful operating points rather than arbitrary thresholds [59,60]. Therefore, evaluating predictive models under equivalent recall–specificity conditions aligns performance assessment with real-world nursing constraints.

From a clinical operations perspective, the observed specificity gains from ML translate into a tangible reduction in unnecessary preventive interventions. This yields less alert fatigue among nursing and clinical staff and better prioritization of borderline-risk patients, focusing their attention on patients most likely to benefit. Furthermore, as tree-based ensemble models are less transparent than rule-based scores, such as the Braden Scale, their reliance on routinely collected nursing data preserves clinical face validity and enables post hoc interpretability analyzes that can be layered in future work.

Regarding the quantification of the operational impact of operating point gains, the relative improvement in specificity is 0.15–0.20 at matched recall (see Figure 4), and the improvement in recall is 0.13–0.25 at matched specificity (see Figure 5). These improvements demonstrate consistent performance gains, and their practical meaning becomes clearer considering the observed PI prevalence of approximately 19% in this cohort. These improvements in the metrics directly imply a substantial reduction in false positive classifications among the majority of patients who will not develop a PI.

In operational terms, this increase in specificity means that a proportion of patients who would have been classified as high risk by the Braden Scale would no longer trigger preventive bundles under the ML model, without increasing the number of missed cases. These avoided false positives correspond to fewer unnecessary repositioning protocols, fewer support surface allocations, or less intensive monitoring plans [4]. In high-volume hospital settings, even moderate reductions in false positives can translate into meaningful decreases in nursing workload and alert fatigue, thereby improving the allocation of preventive resources toward patients.

Conversely, when matching specificity, these gains in recall indicate that a proportion of patients who will develop PI are identified at comparable false-positive burdens. This reflects improved discrimination among truly high-risk individuals without increasing the number of patients classified as high risk. Rather than increasing the preventive workload, the ML model reallocates risk identification toward patients with a higher likelihood of adverse outcomes, enhancing the precision of risk stratification [37].

Therefore, the matched operating-point framework demonstrates that the observed performance differences are not merely statistical improvements in discrimination metrics but reflect tangible operational trade-offs.

Several authors have reported substantial ML gains over traditional risk scores by implicitly comparing models at non-equivalent decision thresholds [14,16]. In such settings, improvements in performance metrics may partly reflect differences in classification policy rather than genuine improvements in discrimination. When an ML model is evaluated at an arbitrary probability threshold, while a clinical instrument is evaluated at a fixed cutoff, the comparison conflates two distinct elements: model discrimination and the chosen operating policy that determines who is classified as high risk.

The matched operating point framework adopted in this paper explicitly separates these dimensions. By constraining recall and specificity to equivalent values across methods, the analysis reduces bias introduced by threshold arbitrariness and ensures that performance differences reflect improvements in discrimination under comparable clinical decision conditions.

This distinction aligns with the literature on prediction model evaluation, particularly papers on threshold probability and decision-curve analysis [59,60]. These frameworks highlight that discrimination metrics do not determine clinical utility. Rather, model performance must be interpreted in the context of the decision threshold at which action is taken. By fixing recall or specificity to clinically meaningful values derived from the Braden Scale, this paper addresses model evaluation under realistic operational constraints.

Although this paper focuses on PI risk assessment and the Braden Scale, the proposed matched operating-point approach is not limited to this clinical context. The same framework can be applied to other screening tools and risk scores widely used in healthcare, enabling fair, clinically grounded comparisons between traditional instruments and ML-based models across a broad range of applications.

The contribution of ML in this paper does not lie in replacing established clinical instruments but in refining risk allocation under fixed operational constraints. By comparing models at matched operating points rather than arbitrary thresholds, this paper shifts the evaluation of clinical prediction from isolated metric optimization toward decision-aligned performance. In this sense, ML enhances precision within existing care structures, supporting more efficient risk stratification without disrupting established clinical workflows.

Overall, the present findings contribute to the growing evidence that ML models can improve PI risk stratification in hospital settings while simultaneously highlighting the continued relevance of traditional nursing assessment tools. Within the current state of knowledge, the results suggest that the most clinically viable strategy may not be the replacement of Braden by ML systems, but rather the integration of both approaches into hybrid decision-support frameworks that combine interpretability, nursing expertise, and data-driven discrimination capability.

## 6. Limitations and Future Research

Although the results are consistent, some limitations must be pointed out. First, the limited sample size constrains the generalizability of the findings to other centers with different typologies, infrastructures, and populations. In this sense, increasing the sample size may enable a better analysis of the effectiveness of traditional scales in different ward units, discovering patterns of performance across various clinical settings. Second, the study is limited to ML models, mainly due to the ease of implementation. Further studies should analyze the performance of more complex ML models, such as heterogeneous stacking models or Deep Learning approaches.

The data come from a single tertiary-level hospital, which may further limit the external validity of the findings in other healthcare institutions with different organizational structures, patient populations, or nursing practices.

The analysis was performed retrospectively using routinely collected nursing records, which are subject to variability, potential measurement error, and missing data. While preprocessing and imputation strategies were applied, residual limitations in data quality may have influenced model performance. In particular, the use of deterministic imputation procedures, including group-wise mean imputation for continuous variables, may have reduced part of the natural variability of some predictors and potentially attenuated underlying relationships between clinical variables and PI occurrence. Although this approach was selected to preserve sample size and maintain clinically relevant observations, alternative imputation strategies should be explored in future studies with larger datasets. This paper focuses on predictive and comparative performance under controlled evaluation conditions and does not assess the prospective clinical impact of implementing ML-based predictions in decision-making processes or PI incidence.

## 7. Conclusions

This study showed that ML models, particularly XGB, consistently outperform the Braden Scale when evaluated under equivalent clinical operating conditions. By applying a matched operating-point framework, the results show that ML models achieve higher discrimination ability, improving either specificity or recall without compromising patient safety. Importantly, the Braden Scale exhibited robust baseline performance, confirming its continued relevance as a conservative and clinically interpretable screening tool in routine nursing practice. As a result, the findings do not suggest that traditional nursing assessment tools should be replaced. Instead, ML models should be viewed as complementary decision-support extensions capable of refining risk stratification, reducing false-positive classifications, and optimizing resource allocation, particularly among borderline-risk patients. In this sense, the most clinically sustainable approach may involve the integration of ML-based models and predictions with established nursing workflows rather than substituting clinical expert judgment or standardized assessment protocols. Overall, these findings highlight the value of clinically grounded evaluation frameworks and support the integration of data-driven tools into nursing workflows to strengthen PI prevention strategies.

## Figures and Tables

**Table 1:** Braden Scale domains and their associated clinical dimensions.

Domain	Measure
Sensory perception	Ability to respond to pressure-related discomfort.
Moisture	Degree to which the skin is exposed to moisture
Activity	Degree of physical activity
Mobility	Ability to change and control body position
Nutrition	Usual food intake pattern
Friction and shear	Degree of assistance needed to move and tendency to slide in bed

**Table 2:** Hyperparameter ranges explored for each machine learning model during the cross-validation optimization process.

Model	Hyperparameter	Values	Models Tested
DT	Splitting criterion	{Gini, Entropy, Log-loss}	1536
	Splitter	{Best, Random}	
	Max. depth	{1, 3, 5, 10}	
	Min. samples to split	{2, 3, 5, 10}	
	Min. samples per leaf	{1, 2, 5, 10}	
	Max. features	{Square root, Logarithmic}	
	Class weights	{None, Balanced}	
LR	Penalty type	{L1, L2}	4000
	Inverse regularization strength (*C*)	from 0.01 to 100 (step: 0.1)	
	Class weights	{None, Balanced}	
RF	Estimators	{10, 50, 100}	2304
	Splitting criterion	{Gini, Entropy, Log-loss}	
	Max. depth	{1, 3, 5, 10}	
	Min. samples to split	{2, 3, 5, 10}	
	Min. samples per leaf	{1, 2, 5, 10}	
	Max. features	{Square root, Logarithmic}	
	Class weights	{None, Balanced}	
XGB	Number of trees	{10, 50, 100}	3840
	Max. depth	{1, 2, 5, 10}	
	Learning rate	from 0.01 to 1.0 (step: 0.05)	
	Subsample ratio	{0.2, 0.5, 0.8, 1.0}	
	Column subsample ratio	{0.1, 0.2, 0.5, 1.0}	
SVC	Kernel	{Linear, RBF}	4000
	Regularization parameter (*C*)	from 0.01 to 10 (step: 0.05)	
	Class weights	{None, Balanced}	

**Table 3:** Mean performance of ML-models in cross-validation and GMS score as overall evaluation metric.

Model	Accuracy	Precision	Recall	Specificity	GMS
DT	79.39%	38.93%	68.40%	75.22%	63.15%
LR	78.65%	44.51%	70.78%	80.09%	66.74%
RF	83.59%	55.10%	58.62%	89.19%	70.05%
XGB	83.60%	56.19%	61.93%	88.31%	71.19%
SVC	77.53%	43.53%	71.40%	78.66%	65.98%

## Data Availability

The data presented in this study are available on request from the corresponding author.

## Supplementary Materials

The following supporting information can be downloaded at: https://www.mdpi.com/article/10.3390/jcm15124683/s1, GMS score and threshold configuration; Table S1: Descriptive statistics of continuous features used as inputs for ML models; Table S2: Descriptive statistics of categorical features used as inputs for ML models; Table S3: Distribution of patients across ward units.

## References

[1] Edsberg L.E., Black J.M., Goldberg M., McNichol L., Moore L., Sieggreen M. (2016). Revised National Pressure Ulcer Advisory Panel Pressure Injury Staging System: Revised Pressure Injury Staging System. J. Wound Ostomy Cont. Nurs..

[2] Li Z., Lin F., Thalib L., Chaboyer W. (2020). Global prevalence and incidence of pressure injuries in hospitalised adult patients: A systematic review and meta-analysis. Int. J. Nurs. Stud..

[3] Alderden J., Cummins M.R., Pepper G.A., Whitney J.D., Zhang Y., Butcher R., Thomas D. (2017). Midrange Braden Subscale Scores Are Associated With Increased Risk for Pressure Injury Development Among Critical Care Patients. J. Wound Ostomy Cont. Nurs..

[4] Padula W.V., Delarmente B.A. (2019). The national cost of hospital-acquired pressure injuries in the United States. Int. Wound J..

[5] Liu S., Rawson H., Islam R.M., Team V. (2024). Impact of pressure injuries on health-related quality of life: A systematic review. Wound Repair Regen..

[6] Padilla P.L., Hancock E.L., Ruff E.S., Zapata-Sirvent R.L., Phillips L.G. (2021). Pressure Injuries. Tips and Tricks in Plastic Surgery.

[7] Weng P., Lin Y., Seo J., Chang W. (2022). Relationship between predisposing and facilitating factors: Does it influence the risk of developing peri-operative pressure injuries?. Int. Wound J..

[8] Kebapci A., Tilki R. (2024). The effect of vasopressor agents on pressure injury development in intensive care patients. Intensive Crit. Care Nurs..

[9] Lachenbruch C., Ribble D., Emmons K., VanGilder C. (2016). Pressure Ulcer Risk in the Incontinent Patient: Analysis of Incontinence and Hospital-Acquired Pressure Ulcers From the International Pressure Ulcer Prevalence™ Survey. J. Wound Ostomy Cont. Nurs..

[10] Coyer F., Cook J.-L., Doubrovsky A., Vann A., McNamara G. (2022). Exploring medical device-related pressure injuries in a single intensive care setting: A longitudinal point prevalence study. Intensive Crit. Care Nurs..

[11] Lima-Serrano M., González-Méndez M., Martín-Castaño C., Alonso-Araujo I., Lima-Rodríguez J. (2018). Predictive validity and reliability of the Braden scale for risk assessment of pressure ulcers in an intensive care unit. Med. Intensiv. (Engl. Ed.).

[12] Jansen R.C.S., Silva K.B.d.A., Moura M.E.S. (2020). Braden Scale in pressure ulcer risk assessment. Rev. Bras. Enferm..

[13] Kwong E., Pang S., Wong T., Ho J., Xue S.-L., Tao L.-J. (2005). Predicting pressure ulcer risk with the modified Braden, Braden, and Norton scales in acute care hospitals in Mainland China. Appl. Nurs. Res..

[14] Zhou Y., Yang X., Ma S., Yuan Y., Yan M. (2022). A systematic review of predictive models for hospital-acquired pressure injury using machine learning. Nurs. Open.

[15] Qu C., Luo W., Zeng Z., Lin X., Gong X., Wang X., Zhang Y., Li Y. (2022). The predictive effect of different machine learning algorithms for pressure injuries in hospitalized patients: A network meta-analyses. Heliyon.

[16] Pei J., Guo X., Tao H., Wei Y., Zhang H., Ma Y., Han L. (2023). Machine learning-based prediction models for pressure injury: A systematic review and meta-analysis. Int. Wound J..

[17] Toffaha K.M., Simsekler M.C.E., Omar M.A. (2023). Leveraging artificial intelligence and decision support systems in hospital-acquired pressure injuries prediction: A comprehensive review. Artif. Intell. Med..

[18] Alves J., Azevedo R., Marques A., Encarnação R., Alves P. (2025). Pressure Injury Prediction in Intensive Care Units Using Artificial Intelligence: A Scoping Review. Nurs. Rep..

[19] Kim M., Kim T.-H., Kim D., Lee D., Kim D., Heo J., Kang S., Ha T., Kim J., Moon D.H. (2024). In-Advance Prediction of Pressure Ulcers via Deep-Learning-Based Robust Missing Value Imputation on Real-Time Intensive Care Variables. J. Clin. Med..

[20] Kim Y., Lim M., Kim S.Y., Kim T.U., Lee S.J., Bok S.-K., Park S., Han Y., Jung H.-Y., Hyun J.K. (2024). Integrated Machine Learning Approach for the Early Prediction of Pressure Ulcers in Spinal Cord Injury Patients. J. Clin. Med..

[21] Nakagami G., Yokota S., Kitamura A., Takahashi T., Morita K., Noguchi H., Ohe K., Sanada H. (2021). Supervised machine learning-based prediction for in-hospital pressure injury development using electronic health records: A retrospective observational cohort study in a university hospital in Japan. Int. J. Nurs. Stud..

[22] Walther F., Heinrich L., Schmitt J., Eberlein-Gonska M., Roessler M. (2022). Prediction of inpatient pressure ulcers based on routine healthcare data using machine learning methodology. Sci. Rep..

[23] Charon C., Wuillemin P.-H., Havreng-Théry C., Belmin J. (2024). One Month Prediction of Pressure Ulcers in Nursing Home Residents with Bayesian Networks. J. Am. Med. Dir. Assoc..

[24] Padula W.V., Armstrong D.G., Pronovost P.J., Saria S. (2024). Predicting pressure injury risk in hospitalised patients using machine learning with electronic health records: A US multilevel cohort study. BMJ Open.

[25] Barriga-Gallegos F., Ríos-Vásquez G., Tapia G.M., Garrido C.A., Vergara N.F. (2026). Early prediction of pressure injury risk in hospitalized patients using supervised machine learning models based on nursing records. Sci. Rep..

[26] Nguyen K.-A., Patel D., Edalati M., Sevillano M., Timsina P., Freeman R., Levin M.A., Reich D.L., Kia A. (2025). Electronic-Medical-Record-Driven Machine Learning Predictive Model for Hospital-Acquired Pressure Injuries: Development and External Validation. J. Clin. Med..

[27] Xu J., Chen D., Deng X., Pan X., Chen Y., Zhuang X., Sun C. (2022). Development and validation of a machine learning algorithm–based risk prediction model of pressure injury in the intensive care unit. Int. Wound J..

[28] Shinkawa M., Mugita Y., Takahashi T., Haba D., Sanada H., Nakagami G. (2025). A novel skin temperature estimation system for predicting pressure injury occurrence based on continuous body sensor data: A pilot study. Clin. Biomech..

[29] Dweekat O.Y., Lam S.S., McGrath L. (2023). An Integrated System of Multifaceted Machine Learning Models to Predict If and When Hospital-Acquired Pressure Injuries (Bedsores) Occur. Int. J. Environ. Res. Public Health.

[30] Ahmed S.R., Kotp M.H., Hafez A.A., Aly M.A., Ismail H.A., Bassiony H.A., Attia A.S., Mekdad A.K., Ahmed R.K. (2025). Nurses’ performance regarding use of Braden scale for predicting pressure ulcers among critically ill patients: Self learning package. BMC Nurs..

[31] Buist M., Bernard S., Nguyen T.V., Moore G., Anderson J. (2004). Association between clinically abnormal observations and subsequent in-hospital mortality: A prospective study. Resuscitation.

[32] Treacy M., Wong G., Odell M., Roberts N. (2022). Understanding the use of the National Early Warning Score 2 in acute care settings: A realist review protocol. BMJ Open.

[33] Talha I., Elkhoudri N., Hilali A. (2024). Major Limitations of Cardiovascular Risk Scores. Cardiovasc. Ther..

[34] Chamnan P., Simmons R.K., Sharp S.J., Griffin S.J., Wareham N.J. (2009). Cardiovascular risk assessment scores for people with diabetes: A systematic review. Diabetologia.

[35] Alemu Y.M., Alemu S.M., Bagheri N., Wangdi K., Chateau D. (2025). Discrimination and calibration performances of non-laboratory-based and laboratory-based cardiovascular risk predictions: A systematic review. Open Heart.

[36] Wei M., Wu L., Chen Y., Fu Q., Chen W., Yang D. (2020). Predictive Validity of the Braden Scale for Pressure Ulcer Risk in Critical Care: A Meta-Analysis. Nurs. Crit. Care.

[37] Huang C., Ma Y., Wang C., Jiang M., Foon L.Y., Lv L., Han L. (2021). Predictive validity of the braden scale for pressure injury risk assessment in adults: A systematic review and meta-analysis. Nurs. Open.

[38] Valiee S., Nemati S.M., Hossaini M., Kashefi H., Mohammadi H. (2022). Comparing the accuracy of the braden and the waterlow scales for pressure ulcer risk assessment in intensive care unit. Nurs. Midwifery Stud..

[39] Park S.-H., Choi Y.-K., Kang C.-B. (2015). Predictive validity of the Braden Scale for pressure ulcer risk in hospitalized patients. J. Tissue Viability.

[40] Timsina P., Joshi H.N., Cheng F.-Y., Kersch I., Wilson S., Colgan C., Freeman R., Reich D.L., Mechanick J., Mazumdar M. (2020). MUST-Plus: A Machine Learning Classifier That Improves Malnutrition Screening in Acute Care Facilities. J. Am. Coll. Nutr..

[41] Sguanci M., Palomares S.M., Cangelosi G., Petrelli F., Sandri E., Ferrara G., Mancin S. (2025). Artificial intelligence in the management of malnutrition in cancer patients: A systematic review. Adv. Nutr..

[42] Hoover P.J., Blumke T.L., Ware A.D., Pillai M., Veigulis Z.P., Curtin C.M., Osborne T.F. (2025). Predicting falls using electronic health records: A time series approach. JAMIA Open.

[43] Shim S., Yu J.Y., Jekal S., Song Y.J., Moon K.T., Lee J.H., Yeom K.M., Park S.H., Cho I.S., Song M.R. (2022). Development and validation of interpretable machine learning models for inpatient fall events and electronic medical record integration. Clin. Exp. Emerg. Med..

[44] Kia A., Timsina P., Joshi H.N., Klang E., Gupta R.R., Freeman R.M., Reich D.L., Tomlinson M.S., Dudley J.T., Kohli-Seth R. (2020). MEWS++: Enhancing the Prediction of Clinical Deterioration in Admitted Patients through a Machine Learning Model. J. Clin. Med..

[45] Muralitharan S., Nelson W., Di S., McGillion M., Devereaux P., Barr N.G., Petch J. (2021). Machine Learning–Based Early Warning Systems for Clinical Deterioration: Systematic Scoping Review. J. Med. Internet Res..

[46] Zheng L., Xue Y.-J., Yuan Z.-N., Xing X.-Z. (2025). Explainable SHAP-XGBoost models for pressure injuries among patients requiring with mechanical ventilation in intensive care unit. Sci. Rep..

[47] Jiang M., Ma Y., Guo S., Jin L., Lv L., Han L., An N. (2021). Using machine learning technologies in pressure injury management: Systematic review. JMIR Med. Inform..

[48] Dweekat O.Y., Lam S.S., McGrath L. (2023). Machine learning techniques, applications, and potential future opportunities in pressure injuries (bedsores) management: A systematic review. Int. J. Environ. Res. Public Health.

[49] Chung M.L., Widdel M., Kirchhoff J., Sellin J., Jelali M., Geiser F., Mücke M., Conrad R. (2022). Risk factors for pressure ulcers in adult patients: A meta-analysis on sociodemographic factors and the Braden scale. J. Clin. Nurs..

[50] Hyun S., Vermillion B., Newton C., Fall M., Li X., Kaewprag P., Moffatt-Bruce S., Lenz E.R. (2013). Predictive Validity of the Braden Scale for Patients in Intensive Care Units. Am. J. Crit. Care.

[51] Rivera-Saba V., Kappes-Ramírez M., Riquelme-Contreras V., Sievers-Frey B., Benavides-Vidal C., Matamala-Troncoso D. (2024). Lesiones por Presión en Ambientes Hospitalarios, un Análisis Regional. Cienc. Enferm..

[52] Ministerio de Salud de Chile (2023). Preguntas y Respuestas: Norma Técnica N°06/2023 Sobre Prevención de Lesiones Por Presión. https://www.minsal.cl/wp-content/uploads/2023/11/PREGUNTAS-Y-RESPUESTAS-LPP-VC-202381747.pdf.

[53] VanGilder C.A., Cox J., Edsberg L.E., Koloms K. (2021). Pressure Injury Prevalence in Acute Care Hospitals with Unit-Specific Analysis: Results from the International Pressure Ulcer Prevalence (IPUP) Survey Database. J. Wound Ostomy Cont. Nurs..

[54] Ríos-Vásquez G., De La Fuente-Mella H., Ceroni-Díaz J. (2025). Group-Specific SVM with Bilevel Programming Methods for Parameter Optimization and Explainable AI in Urban Quality of Life Prediction. IEEE Access.

[55] Pedregosa F., Varoquaux G., Gramfort A., Michel V., Thirion B., Grisel O., Blondel M., Prettenhofer P., Weiss R., Dubourg V. (2011). Scikit-learn: Machine Learning in Python. J. Mach. Learn. Res..

[56] Hyun S., Moffatt-Bruce S., Cooper C., Hixon B., Kaewprag P. (2019). Prediction Model for Hospital-Acquired Pressure Ulcer Development: Retrospective Cohort Study. JMIR Med. Inform..

[57] Dweekat O.Y., Lam S.S., McGrath L. (2022). A Hybrid System of Braden Scale and Machine Learning to Predict Hospital-Acquired Pressure Injuries (Bedsores): A Retrospective Observational Cohort Study. Diagnostics.

[58] Kpatcha E. (2025). Balancing Fairness and Accuracy in Machine Learning-Based Probability of Default Modeling via Threshold Optimization. J. Risk Financ. Manag..

[59] Wynants L., van Smeden M., McLernon D.J., Timmerman D., Steyerberg E.W., Van Calster B. (2019). Three myths about risk thresholds for prediction models. BMC Med..

[60] Vickers A.J., Elkin E.B. (2006). Decision Curve Analysis: A Novel Method for Evaluating Prediction Models. Med. Decis. Mak..

[61] Kennerly S.M., Sharkey P.D., Horn S.D., Alderden J., Yap T.L. (2022). Nursing Assessment of Pressure Injury Risk with the Braden Scale Validated against Sensor-Based Measurement of Movement. Healthcare.

[62] Susanto A.P., Lyell D., Widyantoro B., Berkovsky S., Magrabi F. (2023). Effects of machine learning-based clinical decision support systems on decision-making, care delivery, and patient outcomes: A scoping review. J. Am. Med. Inform. Assoc..

[63] Sokol K., Fackler J., Vogt J.E. (2025). Artificial intelligence should genuinely support clinical reasoning and decision making to bridge the translational gap. npj Digit. Med..

