# Supplementary material for: Pressure Injury Risk Assessment in Nursing Practice: A Head-to-Head Comparison of the Braden Scale and Machine Learning Models
Source: J Clin Med. 2026 Jun 17;15(12):4683. doi: 10.3390/jcm15124683 (PMC13301824; doi:10.3390/jcm15124683)
Supplement: Supplementary file 1 [file jcm-15-04683-s001.zip › jcm-4277305-supplementary.pdf]

Supplementary Material – GMS score and threshold configuration

Let's consider $\mathcal{M}_{ij}$ a performance metric, such that $j \in J$, where J is the set of metrics calculated for a model i. The geometric mean score (GMS) is given by Equation (S1).

$$GMS_i = \sqrt[|J|]{\prod_{j=1}^{J} M_{ij}} \quad \text{(S1)}$$

The output probability that an instance belongs to a particular class is denoted by $\hat{p}(\mathbf{x})$, where $\mathbf{x}$ is the set of features used to calculate the probability (risk), formally it can be expressed as shown in Equation (S2).

$$\hat{p}(\mathbf{x}) = \mathbb{P}(y = 1|\mathbf{x}) \quad (S2)$$

Once $\hat{p}(\mathbf{x})$ is computed, binary class labels can be obtained by applying a decision threshold $h \in [0,1]$ to $\hat{p}(\cdot)$. This means that any value of $\hat{p}(\cdot) > h \rightarrow y = 1$ while otherwise its value is 0.

Supplementary Material – Table S1: Descriptive statistics of continuous features used as inputs for ML models

Variable	Mean	Min	Max
Age	47	1	99
Waiting days	10	0	30
Height (cm)	138	46	182
Weight (kg)	62.1	3.1	160.0

Supplementary Material – Table S3: Distribution of patients across ward units

Service	N	Share
Adult Surgical Medical Unit	187	42.0%
Adult surgery	52	11.7%
Emergency Department	36	8.1%
Adult intermediate care	33	7.4%
Adult intensive care	25	5.6%
Pediatric surgery	21	4.7%
Pediatric medicine	21	4.7%
Pediatric intermediate care	18	4.0%
Pediatric surgical unit	13	2.9%
Neonatal intermediate care	12	2.7%
Neonatal intensive care	9	2.0%
Gynecology	8	1.8%
Neonatology	7	1.6%
Recovery	3	0.6%

Supplementary Material – Table S2: Descriptive statistics of categorical features used as inputs for ML models

Variable	Group	N	Share
Gender	Male	243	54.6%
	Female	202	45.4%
Dependency risk	High risk (total dependency)	109	24.8%
	Medium risk (total dependency)	89	20.3%
	Maximum risk (total dependency)	75	17.1%
	Medium risk (partial dependency)	70	15.9%
	Medium risk (self-sufficiency)	32	7.3%
	High risk (partial dependency)	27	6.2%
	Low risk (partial dependency)	21	4.8%
	High risk (partial dependency)	5	1.1%
	High risk (self-sufficiency)	5	1.1%
	Low risk (total dependency)	4	0.9%
	Low risk (partial dependency)	2	0.4%
Mattress	Yes	193	43.4%
	No	252	56.6%
Position assistance	Yes	274	61.6%
	No	193	38.4%
Skin treatments	Yes	256	57.5%
	No	189	42.5%
Nutrition	No	156	35.1%
	Yes	289	64.9%
Neuromuscular	Yes	11	2.5%
	No	434	97.5%
Mobility	Yes	35	7.9%
	No	410	92.1%
Restraints	Yes	58	13.1%
	No	387	86.9%
Humidity	Yes	34	7.7%
	No	411	92.3%
Incontinence	Yes	167	37.5%
	No	278	62.5%
Incontinence type	No incontinence	278	62.5%
	Mixed	132	29.7%
	Urine	22	4.9%
	Fecal	13	2.9%
Skin lesions	Yes	29	6.5%
	No	416	93.5%
Medical devices	Yes	166	62.7%
	No	279	37.3%
Invasive devices	Yes	306	68.7%
	No	139	31.3%
Recent surgery	Yes	101	22.7%
	No	344	77.3%
PI pre-hospital	Yes	24	5.4%
	No	421	94.6%
